# DISCRIMINATION, STRESS, AND HEALTH ACROSS THE LIFE COURSE

**DOI:** 10.1093/geroni/igz038.687

**Published:** 2019-11-08

**Authors:** Roland J Thorpe, Carl V Hill

**Affiliations:** 1 Johns Hopkins Bloomberg School of Public Health, Baltimore, Maryland, United States; 2 NIH/National Institute on Aging, Bethesda, Maryland, United States

## Abstract

There is a paucity of research that seeks to understand why race disparities in health across the life course remain elusive. Two such explanations that have been garnering attention is stress and discrimination. This symposium contains papers seeking to address the impact of discrimination or stress on health or health disparities across the life course. First, Brown and colleagues examine black-white differences in the number of reported chronic stressors across five domains their appraised stressfulness, and their varying associations with anxiety and depression among a diverse sample of older adults using data from 6,019 adults ages 52+ from the 2006 HRS. Race and appraisal interactions show that blacks and whites report similar increases in anxiety and depressive symptoms with appraisal. Second, Tobin and colleagues investigate the impact of early life racial discrimination (ELRD) on mental health among Black adults. Using data from 618 participants in the Nashville Stress and Health Study, these investigators found that childhood and adolescent ELRD were positively associated with adult distress. Also, individuals who experienced childhood ERLD had 88% lower odds of adult MDD than individuals with no ELRD. Cobb and colleagues examine the cross-sectional association between everyday discrimination and kidney function among older adults in HRS. The authors report that after adjusting for demographic characteristics, everyday discrimination was associated with lower mean eGFR. The relationship between everyday discrimination and kidney function was not explained by biospsychosocial factors. This collection of papers provides insights into how discrimination or stress impacts health in middle to late life.

